# Global variations in oncology professionals’ confidence levels for managing antibody–drug conjugate toxicities: a cross-continental survey

**DOI:** 10.1093/oncolo/oyaf232

**Published:** 2025-07-28

**Authors:** Marina Campione, Michele Maffezzoli, Aruni Ghose, Akash Maniam, Ria Nagpal, Pasquale Rescigno, Sebastiano Buti, Renita George, Kevin Bambury, Richard Bambury, Eoin O’Carroll, Giuseppe Luigi Banna

**Affiliations:** Faculty of Economics and Law of University of Enna Kore, Enna 94100, Italy; Portsmouth Hospitals University NHS Trust, Portsmouth PO6 3LY, United Kingdom; Medical Oncology Unit, University Hospital of Parma, Parma, Italy; Department of Medicine and Surgery, University of Parma, Parma, Italy; Barts Cancer Centre, St Bartholomew’s Hospital, Barts Health NHS Trust, London, United Kingdom; Portsmouth Hospitals University NHS Trust, Portsmouth PO6 3LY, United Kingdom; Portsmouth Hospitals University NHS Trust, Portsmouth PO6 3LY, United Kingdom; Translational and Clinical Research Institute, Centre for Cancer, Newcastle University, Newcastle upon Tyne, United Kingdom; Medical Oncology Unit, University Hospital of Parma, Parma, Italy; ONCOassist, Ireland; ONCOassist, Ireland; ONCOassist, Ireland; CUH/UCC Cancer Centre, Cork University Hospital, Cork, Ireland; ONCOassist, Ireland; Portsmouth Hospitals University NHS Trust, Portsmouth PO6 3LY, United Kingdom; Faculty of Science & Health, School of Pharmacy & Biomedical Sciences, University of Portsmouth, Portsmouth PO1 2UP, United Kingdom

**Keywords:** ADC, toxicity, adverse events, cancer, survey, app

## Abstract

**Background:**

Antibody–drug conjugates (ADCs) represent a promising therapeutic approach in oncology, but managing their toxicities remains challenging. This survey aimed to assess confidence levels in ADC toxicity management among oncology professionals globally.

**Methods:**

A cross-sectional survey was conducted using the ONCOassist mobile, a CE-marked oncology application widely used by oncologists globally as support in clinical practice. Confidence in toxicity management was measured on a 5-point scale. Predictor variables included income level, professional role, specialization, and cancer type treated.

**Results:**

The survey was offered to 5883 users, and responses were received from 1256 oncology healthcare professionals (HCPs) across various continents and professional roles. Medical oncologists represented the largest group (46%). Most of the HCPs were from high- or upper-middle-income countries (75%) with most nurses (86%) from high-income countries. Respondents from Europe and North America reported higher confidence ratings, while those from Asia, Africa, and Oceania tended to report lower confidence levels (*P* < .001). Respondents from lower-middle-income countries reported lower confidence compared with those from high- and upper-middle-income countries (*P* < .001). Additionally, senior physicians exhibited substantially higher confidence compared to junior physicians and nurse practitioners (*P* < .001, for both). Specialty affected confidence levels, particularly in surgical oncologists who reported the lowest confidence (*P* < .001).

**Conclusion:**

This global survey reveals significant disparities in confidence levels for managing ADC toxicities across regions, professional roles, and specializations. These findings may suggest the need for targeted educational interventions and support systems to enhance competences in managing ADC-related toxicities, ultimately improving the global standard of care for patients receiving ADCs.

Implications for Practice This survey aimed to assess global variations in oncology professionals’ confidence in managing antibody–drug conjugate (ADC) toxicities, identifying disparities across regions, roles, and specializations. 1256 oncology healthcare professionals from various continents responded to the survey. This study revealed significant geographical and professional disparities in ADC toxicity management confidence. Understanding these differences can help implement targeted educational interventions to improve ADC toxicity management and, ultimately, provide better care for patients.

## Introduction

Antibody–drug conjugates (ADCs) represent promising drugs in oncology, offering targeted treatment with potentially reduced systemic toxicity.^1^ Since its first description in the 1960s, this space has seen remarkable growth, with 11 FDA-­approved ADCs and more than 210 in the pipeline.^2^

While conventional cytotoxic chemotherapy has been crucial in improving survival rates for many cancer types, their efficacy is often limited by narrow therapeutic windows. ADCs address these limitations by expanding these therapeutic indices.^3^ They consist of a tumor-targeting monoclonal antibody (mAb) conjugated to an active chemotherapeutic molecule via a linker, combining the highly specificity of mAbs with the extremely potent cytotoxic effect of the chemotherapeutic agent, therefore minimizing off-target toxicity.^4^

However, toxicities can still occur, either similar to and distinct from those associated with traditional chemotherapy, often tied to the drug-linker component rather than the antibody or target. Notable instances are hematotoxicity, hepatotoxicity, and gastrointestinal reactions.^5^ This off-target toxicity may stem from premature release of cytotoxic payloads into circulation or internalization of the drug even in the absence of target receptors.^6^^,^^7^ Additional potential mechanisms could be immune-mediated toxicity through the interaction of the immune system with the Fc portion of ADCs (via ADCC and ADCP), endoplasmic reticulum stress, and immune cell recruitment and activation.^8^^,^^9^

The management of ADC toxicities remains a critical challenge for healthcare professionals (HCPs) worldwide. As the field rapidly expands, the complexity of managing ADC-related adverse events has increased. A review of 169 trials found 600 different adverse events for ADCs, with a treatment-related discontinuation rate of 13.2% and a rate of any grade treatment-related adverse events of 91.2%.^5^ To address these challenges, experts recommend a multidisciplinary approach to manage ADC-related toxicities, similar to the strategies developed for immunotherapy.^5^^,^^10^

In this context, we performed a survey aimed to assess the confidence levels in toxicity management among oncology professionals across different continents, roles, and specializations. Understanding these confidence patterns is crucial for identifying areas, where additional support and education may be needed to optimize patient care in the evolving landscape of ADC therapies. This assessment is particularly timely given the rapid expansion of ADCs in clinical practice, both as monotherapy and in combination with other agents (such as immunotherapy), as well as the ongoing challenges in predicting their activity and effectiveness, optimizing their sequencing, and minimizing their side effects.^11^

## Methods

### Study design and participants

This global cross-sectional survey assessed confidence levels in toxicity management among HCPs involved in cancer care across various continents, professional roles, and specializations. The study utilized the ONCOassist mobile application (Figure S1), a CE-approved oncology clinical decision support tool (https://wwwemaeuropaeu/en/human-regulatory/overview/medical-devices), to distribute the survey. ONCOassist mobile application is widely used oncology clinical decision support tool that is constantly improving based on user feedback. Since it was originally developed in University College Cork through the Masters in E-business program in 2012, ONCOassist has received wide-scale acceptance amongst oncology clinicians globally. It was promoted by the European Society of Medical Oncology in 2015 and is now used in more than 180 countries worldwide. A study carried out and published about its adoption by clinicians throughout Europe in 2019 also describes the process it uses to engage with users and improve its usability.^12^

Participants included physicians and nurses involved in cancer treatment across various continents and specializations. The survey was conducted between July 1, 2024 and August 28, 2024, via an in-app survey, which was distributed to ONCOassist users globally. Two separate surveys were sent, the first to physicians and the second to oncology nurses. HCPs were defined as physicians (senior and junior) and nurses. At the time of distribution, the ONCOassist community comprised approximately 88 000 members. The questionnaire was sent in English via a push notification and pop up. The survey reached 5883 members. Not all users received the survey due to-opt-outs, filtering criteria for HCPs, or recent contact from ONCOassist. Participants only received the survey once.

The primary outcome variable was confidence in toxicity management, measured on an ordinal scale from 1 (lowest) to 5 (highest). Predictor variables included (1) income level, categorized based on World Bank country economic classifications for 2024-2025; (2) continent: Africa, Asia, Europe, North America, South America, and Oceania; (3) role: senior physician, junior physician, nurse, and nurse practitioner; (4) specialization: hematology, medical oncology, radiation/clinical oncology, and surgical oncology; and (5) cancer type: breast, gastrointestinal, genitourinary, gynecologic, head and neck, lung, and lymphoma.

### Survey instrument

The questionnaire was developed by G.L.B., K.B., R.G., and E.O.C., designed to evaluate participants’ confidence in managing ADC-related toxicities. The questionnaire consisted of 1 question (Figure S2). Respondents were asked: “On a scale from 1 (not at all confident) to 5 (extremely confident), how would you rate your confidence in managing ADC-related toxicities in your clinical practice?”. Single-item confidence measures were selected based on evidence supporting their validity for global assessments in different settings and to minimize response burden in this multinational study.^13–17^ Demographic and professional information were collected including the participant’s continent, professional role, and oncology specialization via the user’s profile. Demographic and professional information—including continent, profession, and oncology specialization—was extracted from ONCOassist user profiles, where these details were self-reported during the registration process (Figure S3).

### Data collection

Data were collected over 1 month through the ONCOassist app using intercom in the app survey feature (https://www.intercom.com/drlp/surveys). Participation was voluntary. continents were defined by the United Nations M49 Standard Division UNS. United nations m49 standard country or area codes for statistical use (https://unstatsunorg/unsd/methodology/m49/2021; Series M). Countries have been divided into income groups according to the most recent data from the World Bank’s income classification, which categorizes nations based on their Gross National Income per capita. These groups—high-income, upper-middle-income, lower-middle-income, and low-income—are updated annually by the World Bank to reflect changes in income levels worldwide. The classifications used in this analysis aligns with the World Bank’s latest updates for the fiscal year 2024-2025 (https://datahelpdesk.worldbank.org/knowledgebase/articles/906519).

### Statistical analysis

Descriptive statistics (eg, frequencies, means, and medians) were used to summarize participants’ demographic characteristics and their self-reported confidence in managing ADC-­related toxicities. Differences in confidence levels across continents, professional roles, and specializations were examined using analysis of variance for parametric comparisons and the Kruskal–Wallis test for nonparametric comparisons, with statistical significance set at *P < *.05. The ordinal nature of the outcome variable made it suitable for analysis using cumulative logit modelling, allowing to evaluate factors associated with varying levels of confidence. Because the primary outcome—self-reported confidence—was measured on an ordinal scale from 1 (lowest) to 5 (highest), we applied cumulative logit modelling (proportional odds) to evaluate factors associated with higher confidence. Predictor variables included country income classification (low-, lower-middle-, upper-middle-, and high-income), continent (Africa, Asia, Europe, North America, South America, and Oceania), professional role (senior physician, junior physician, nurse, and nurse practitioner), specialization (hematology, medical oncology, radiation/clinical oncology, and surgical oncology), and cancer type (breast, gastrointestinal, genitourinary, gynecologic, head and neck, lung, and lymphoma).

The statistical analysis proceeded in 2 stages. First, univariate cumulative logit models were fitted for each predictor, yielding unadjusted odds ratios (OR) and 95% CI. Predictors meeting the significance threshold (*P < *.05) or deemed clinically important were then entered into a multivariate cumulative logit model. The proportional odds assumption was evaluated via a likelihood-ratio test, and model fit was assessed by comparing Akaike Information Criterion values. ORs were obtained by exponentiating the model coefficients, where values >1 indicate greater odds of reporting higher confidence and values <1 indicate lower odds. All analyses were conducted using R version 4.4.0., with cumulative logit models fitted using the “ordinal” package in R.

### Ethical considerations

Ethical committee approval and consent were not sought due to the minimal risk to individuals, as no intervention or identifiable private information was obtained. The survey aimed to identify areas for improvement within the ONCOassist community.

## Results

### Characteristics of oncology professionals

Our global survey gathered responses from a total of 1256 oncology HCPs, comprising 1023 physicians and 233 nurses. The detailed breakdown and definition of respondents’ countries by continents, country income levels, and their specific roles are presented in Table S1.

Medical oncologists formed the largest group, representing 46% of the specialty category, followed by clinical and radiation oncologists (26%) and medical oncology nurses (9%). Smaller groups included hematologists (4%) and hematology nurses (3%). In terms of job type, physicians constituted the majority at 81%, while nurses accounted for 16%. Among physicians, 54% were senior physicians, and 47% were junior physicians. For nurses, 70% were general nurses, and 30% were nurse practitioners, indicating a significant presence of specialized and advanced nursing roles (Table S2).

The respondents were primarily involved in managing patients with gastrointestinal (37%), breast (31%), and lung cancer (11%). A similar distribution pattern was observed between physicians and nurses across most categories (Table S3).

Most participants were from high- or upper-middle-income countries (75%). In high-income countries, respondents constituted 47% of the total (including 38% and 86% of the total physicians and nurses); in upper-middle income countries, respondents constituted 28% of the total (33% and 9% of the total physicians and nurses), while lower-middle-income countries accounted for 24% (28% and 5% of the total physicians and nurses). The representation from low-income countries was minimal (less than 1%). This distribution suggests a stronger representation from higher-income countries, particularly among nursing staff (Table S4).

Geographically, the survey saw a higher concentration of respondents from Europe (37%) and Asia (27%). Notably, there was a high representation of nurses in Europe and North America, indicating geographic variations in survey participation (Table S5).

### Geographical variations in confidence levels

**Figure 1.**
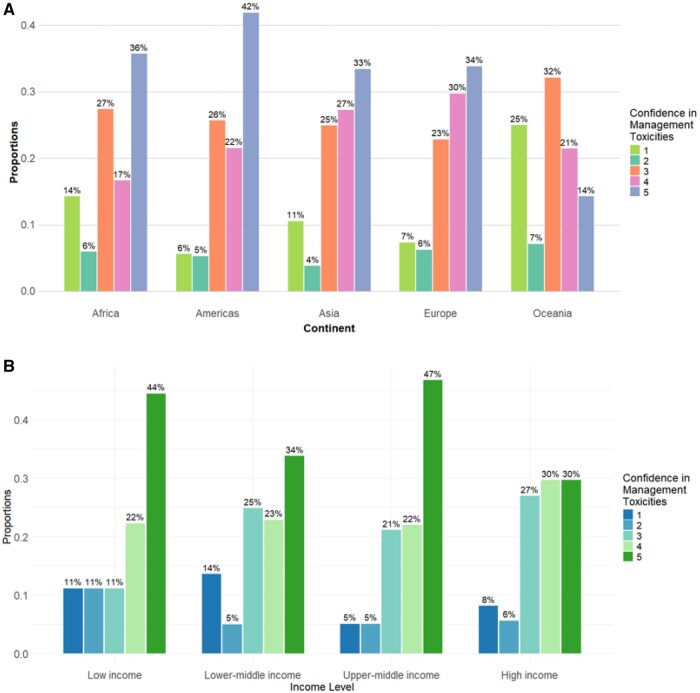
Confidence in management toxicities among survey respondents by continent (A) and country income level (B).

Analysis of confidence levels in managing ADC toxicities showed notable differences among physicians and nurses across continents and income levels. For physicians, confidence levels vary significantly across both continents and income levels (*P < *.001 for both) (Tables 1 and 2). Higher confidence scores (4 and 5) were more prevalent in European (29% and 37%, respectively) and American oncology physicians (21% and 44%), compared to Asian (28% and 33%), African (15% and 35%), and Oceanian (21% and 5%) colleagues (*P < *.001) (Figure 1A and Figure S4). The lowest confidence levels (1 and 2) were reported by oncology physicians from lower-middle (13% and 5%) compared to high- (7% and 4%) and upper-middle- (5% and 5%) income countries (*P < *.001) (Figure 1B).

**Table 1.** Confidence in management toxicities among physicians by continent.

Confidence	Africa (79)	%	Asia (326)	%	Europe (349)	%	America (250)	%	Oceania (19)	%	*P*-value
1	12	15.19	33	10.12	21	6.02	15	6	4	21.05	
2	5	6.33	13	3.99	16	4.58	14	5.6	1	5.26	*<* **.001**
3	22	27.85	82	25.15	82	23.50	58	23.2	9	47.37	
4	12	15.19	92	28.22	102	29.23	54	21.6	4	21.05	
5	28	35.44	106	32.52	128	36.68	109	43.6	1	5.26	

**Table 2.** Confidence in management toxicities among physicians by country income level.

Confidence	Low (7)	%	Lower-middle (291)	%	Upper-middle (335)	%	High (390)	%	*P*-value
1	1	14.29	38	13.06	18	5.37	28	7.18	
2	1	14.29	15	5.15	18	5.37	15	3.85	*<* **.001**
3	1	14.29	74	25.43	71	21.19	107	27.44	
4	1	14.29	69	23.71	76	22.69	118	30.26	
5	3	42.86	95	32.65	152	45.37	122	31.28	

For nurses, the confidence levels also differed across continents and income levels (*P = *.02 and <.001, respectively) (Tables S6 and S7). Higher confidence scores (4 and 5) were more prevalent in African oncology nurses (40% and 40%) compared to the American (21% and 37%), European (31% and 25%), Asian (7% and 53%), and Oceanian (22% and 33%) colleagues (*P = *.02) (Figure 1A and Figure S4). Most of the oncology nurses were from high-income countries (86%, 200 out of 233) with only a minority of them reporting the lowest confidence levels (10% and 9%, confidence levels 1 and 2) (Figure 1B).

### Confidence levels by professional role and experience

**Figure 2.**
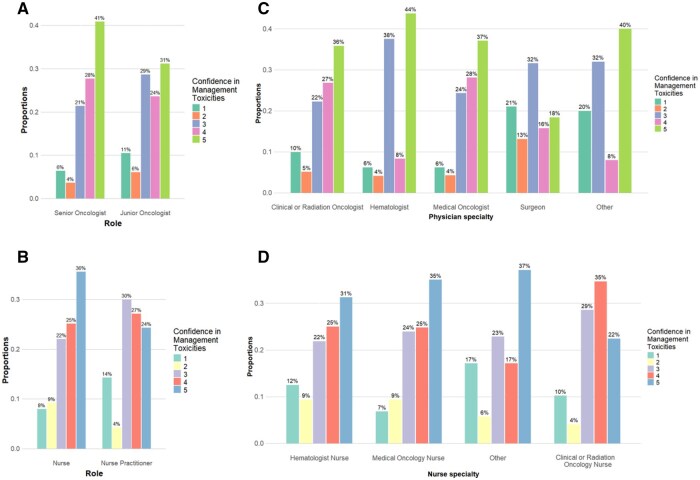
Confidence in management toxicities among survey respondents by physician (A) and nurse role (B) and physician (C) and nurse (D) speciality.

Significant differences in confidence levels among physicians by level of experience were found (*P < *.001) (Table 3). Higher confidence levels (4 and 5) were reported by senior physicians (28% and 41%) compared to junior physicians (24% and 31%), suggesting that experience impacts on the confidence in managing ADC-related toxicities. There were no statistically significant differences in confidence levels between general nurses and nurse practitioners (*P = *.11) (Figure 2A and Table S8).

**Table 3.** Confidence in management toxicities among senior and junior physicians.

Confidence	Senior physician	%	Junior physician	%	*P*-value
1	35	6.38	50	10.52	
2	20	3.65	29	6.10	
3	117	21.35	136	28.63	*<* **.001**
4	152	27.73	112	23.58	
5	224	40.87	148	31.16	

No significant differences in overall confidence levels were observed between physicians and nurses, with both groups showing a comparable level of confidence in ADC management (*P = *.77) (Figure 2B and Table S9).

Confidence levels varied significantly across physician specializations (*P = *.002), with the highest confidence levels (4 and 5) primarily found among medical oncologists (28% and 37%) and clinical and radiation oncologists (26% and 35%), followed by hematologists (8% and 44%) and surgeons (16% and 18%) (Table 4 and Figure 2C). Of note, hematologists demonstrated the highest confidence (44% level 5), despite comprising only 3.8% of respondents (*n* = 48).

**Table 4.** Confidence in management toxicities among survey respondents by physician specialization.

Confidence	Clinical and radiation	%	Hematologist	%	Medical	%	Surgeon	%	Other	%	*P*-value
1	33	9.94	3	6.25	36	6.21	8	21.05	5	20.00	
2	17	5.12	2	4.17	25	4.31	5	13.16	0	0.00	
3	74	22.29	18	37.50	141	24.31	12	31.58	8	32.00	**.002**
4	89	26.81	4	8.33	163	28.10	6	15.79	2	8.00	
5	119	35.84	21	43.75	215	37.07	7	18.42	10	40.00	

No significant variation in confidence levels was seen across nurse specializations (*P = *0.79) (Figure 2D and Table S10).

Additionally, confidence levels did not vary significantly across different cancer types (*P = *.4483), suggesting a similar confidence in ADC toxicity management across sub-specializations and implying that HCPs share similar confidence levels in handling ADC-related toxicities, irrespectively of their specific cancer focus (Table S11**)**. A higher confidence level was reported for gastrointestinal (GI) cancers (38% level 5), lung cancers (35% level 5), and breast cancers (35% level 5).

### Univariate and multivariate analysis

The univariate and multivariate analyses revealed significant factors influencing HCPs confidence in managing ADC toxicities in toxicity management according to their experience, specialization, and regional factors. Income level showed a nonlinear relationship with confidence (*P < *.001). Geographically, South American professionals demonstrate higher confidence levels (*P < *.001), while those in Oceania report lower levels (*P = *.01) compared to other continents. Role-based differences were evident, with junior physicians (*P < *.001) and nurse practitioners (*P < *.001) reporting significantly lower confidence than senior physicians. Specialization impacted confidence levels: surgical oncologists reported the lowest confidence (*P < *.001), while medical oncologists had 3.9 higher odds of confidence than surgeons (OR 3.92, *P *< .001) in multivariate analysis (Table 5). The Brant test indicated no violation of the proportional odds assumption (>.5).

**Table 5.** Univariate and multivariate analysis on variations in HCPs confidence in toxicity management.

	Univariate analysis				Multivariate analysis			
Coefficient	Estimate	SE	OR	Pr(>|z|)	Estimate	SE	OR	Pr(>|z|)
**Income level**								
Income level linear effect	0.03	0.09	1.03	.766				
Income level quadratic effect	−0.47	0.09	0.63	**<.001**				
**Continent**								
North America (Baseline)	1				1			
Africa	−0.16	0.25	0.85	.52	−0.11	0.26	0.89	.66
Asia	0.03	0.17	1.03	.86	0.10	0.19	1.11	.59
Europe	0.11	0.16	1.11	.51	0.04	0.17	1.04	.81
Oceania	−1.03	0.37	0.36	**.01**	−1.04	0.37	0.35	**.01**
South America	0.62	0.20	1.86	**.02**	0.67	0.21	1.96	**.01**
**Role**								
Senior physician (baseline)	1.00				1			
Junior physician	−0.51	0.11	0.60	**<.001**	−0.51	0.12	0.60	**<.001**
Nurse	−0.31	0.16	0.73	**.05**	−0.25	0.17	0.78	.14
Nurse practitioner	−0.73	0.23	0.48	**<.001**	−0.70	0.24	0.50	**<.001**
**Specialization**								
Surgical oncology (Baseline)	1.00				1.00			
Medical oncology	1.20	0.30	3.32	**.03**	1.37	0.30	3.92	**<.001**
Radiation or clinical oncology	1.06	0.31	2.89	**<.001**	1.19	0.31	3.29	**<.001**
Hematologist	1.02	0.36	2.77	**.05**	1.22	0.36	3.40	**<.001**
**Cancer type**								
Breast cancer (baseline)	1.00							
Gastrointestinal cancers	0.12	0.12	1.12	.36				
Genitourinary cancers	−0.02	0.29	0.98	.95				
Gynecologic cancers	0.08	0.33	1.08	.78				
Head and neck cancer	−0.51	0.24	0.60	**.03**				
Lung cancer	0.08	0.18	1.08	.66				
Lymphoma	−0.36	0.31	0.70	.24				

## Discussion

Our findings reveal significant variations in confidence levels for ADC toxicity management across geographical regions, professional roles, and oncology specializations. These variations underscore the complex landscape of ADC therapy implementation and the challenges faced by HCPs globally.

The geographic disparity may reflect differences in healthcare systems, reimbursements, resources, and cultural factors, influencing confidence in toxicity management. Country income level showed a nonlinear relationship with confidence, with respondents from lower-middle-income countries reporting lower confidence compared with those from high- and upper-middle-income countries. The higher confidence levels in South America are particularly interesting, given that ADCs are relatively new to this region. This could indicate a more optimistic approach to novel therapies or potentially a gap between perceived and actual competence that warrants further investigation.

The study highlighted a clear hierarchy in confidence levels based on professional experience, with junior physicians and nurse practitioners reporting significantly lower confidence compared to senior physicians. This trend underscores the importance of experience in building confidence for managing complex ADC-related toxicities. The lower confidence levels among junior professionals and the impact of the experience in managing novel therapies align with previous studies.^18–20^ This finding emphasizes the need for targeted training programs and mentorship opportunities to bridge the confidence gap between junior and senior oncology professionals.^21^

Notably, surgical oncologists exhibited the lowest confidence levels among specializations. This could be attributed to the fact that ADCs are primarily used in oncology and hematology, and surgical oncologists may have less direct experience with cytotoxic agents. The exact ADC usage rates by specialty were not collected and is currently unavailable, limiting our ability to quantitatively link confidence to ADC usage rates by specialty. However, usage patterns can be broadly inferred based on known approvals. As of 2024, 13 ADCs have been approved, 7 in hematology and 6 in oncology, with 4 approved for breast cancer.^22^ Recent meta-analyses demonstrated that 91.2% of ADC-treated patients experience any-grade treatment-related adverse events (TRAEs), with 13.2% discontinuing treatment due to toxicity, reflecting how common ADC-related toxicities are in oncology and hematology practice.^5^ Hematologists’ high confidence (44% at level 5) may reflect their focused experience with ADCs that have been approved and used for a long time, such as brentuximab vedotin.^22^ The lack of confidence variation by cancer type suggests that ADC experience may transcend tumor-specific practice patterns. Breast cancer specialists showed a high confidence level in managing ADC-related toxicities, which is expected given that this is the specialty where ADCs have been used most extensively and for the longest time in oncology. A recent meta-analysis showed that, in patients with metastatic breast cancer, the prevalence of the most common TRAEs ranged from 12% to 33%, depending on the ADC type and study design. Gastrointestinal disorders were highly prevalent with trastuzumab deruxtecan, general disorders were common with trastuzumab emtansine, while hematologic and gastrointestinal toxicities were most frequently reported with sacituzumab govitecan.^23^ Notably, breast cancer specialists had comparable confidence levels to GI and lung cancer specialists, despite differing ADC usage. This could reflect the increasing use of ADCs in GI and lung cancer clinical trials, where ADC development has been rapidly expanding.^24^^,^^25^ However, this apparent uniformity should be interpreted with caution, as confidence was self-reported and not directly linked to the type, number, or geographic availability of approved ADCs, which can substantially influence personal expertise. Future interdisciplinary collaborations could help fill the gap between specialties and sub-specialties, ensuring more comprehensive patient care in managing ADC-related toxicities.

The observed confidence variations have significant implications for the global implementation of ADC therapies. Confidence in toxicity management is crucial for the optimal use of these agents, as distrust may lead to underutilization, while overconfidence could result in inadequate toxicity monitoring.^26^ These disparities in confidence levels suggest that the adoption and effective use of ADCs may differ considerably across different regions and healthcare settings.

These results emphasize the need for targeted educational interventions and support systems tailored to specific regions, roles, and specializations to enhance overall confidence and competence in managing ADC-related toxicities. Continuous medical education programs, international and multidisciplinary collaborations, and the development of standardized guidelines for ADC toxicity management could help address these disparities. Additionally, leveraging technology for remote learning and consultation could be particularly beneficial for regions with lower confidence levels. Finally, considering the broad spectrum of ADC-related toxicities, easy access to multidisciplinary management could be a key factor influencing clinicians’ use and confidence. The spectrum of toxicities varies widely across ADC compounds in accordance with their different cytotoxic payloads and target molecules. This poses an additional challenge for HCPs and for CME efforts, unlike other drug classes such as immune checkpoint inhibitors, which have broadly similar toxicity profile.

While our study provides valuable insights into the confidence level of oncology professionals in managing ADC-related toxicities, several limitations should be considered when interpreting the results.

The single-item assessment of confidence represents the most important limitation of this study. While this approach enabled broad participation across diverse healthcare settings and countries, it necessarily restricted the depth of our analysis. The single-item approach, which has been validated in various oncology settings, was deliberately chosen to also maximize response rates across our global survey.^14^^,^^16^^,^^17^ This design allowed us to capture a broad snapshot of confidence levels across more than 180 countries.

The study relies on self-reported confidence levels, which may not accurately reflect actual clinical competence or performance in managing ADC toxicities. While correlating confidence with objective toxicity data would be ideal, such analysis was beyond the scope of this survey due to the lack of standardized, publicly available reporting systems across the diverse countries included. Future studies could benefit from incorporating objective measures of competence and real-world toxicity metrics through multicenter international collaborations.

Our survey included respondents from low-income countries, although they represented less than 1% of the total sample, which may reflect a limited access to new expensive drugs like ADCs in these regions. This small subgroup size precludes robust statistical comparisons and limits generalizability to a broader oncology community. Moreover, as the survey was distributed through the ONCOassist mobile application, our sample may be biased toward HCPs who are more technologically inclined or have access to such resources, further limiting participation from lower-income countries. To address the disparities in ADC access and expertise across low-income countries, a coordinated global strategy is needed. Potential strategies may include a global access program for ADCs, partnerships with high-volume centers in high-income countries, and global digital training initiatives to support local HCPs in safely using and managing ADCs.

The cross-sectional nature of our study provides a snapshot of confidence levels at a single point in time. Longitudinal studies would be valuable to track how confidence levels change as ADCs become more established in clinical practice. Specifically, several approaches can generate high-level of evidence: global surveys balanced to include objective measures of ADC exposure, prospective multicenter toxicity registries and trials testing targeted training interventions.

Our survey did not capture potentially important contextual factors, including institutional support, frequency of ADC use in clinical practice, variations in following institutional and international guidelines for ADC toxicity management, and availability of training and educational resources. While such contextual factors likely contribute to confidence disparities, systematic data on guideline adherence, and training standards across all included countries are not available. Furthermore, clinical practice patterns for ADC management often vary substantially even within countries and centers, depending on institutional resources and individual clinician experience. These factors could influence confidence levels and provide a more comprehensive understanding of the observed variations. Future studies could benefit from incorporating questions about guideline use and training exposure to better understand the drivers of confidence variations.

While the study provides insights across continents, it does not account for potential variations within countries or regions in ADC approval status and duration of clinical use. Additionally, we did not control for potential confounding factors such as years of experience within each professional role or the specific types of ADCs used in different regions and sub-­specialties. All these factors could significantly influence confidence levels. To address these gaps, future studies should encourage international collaborations to capture diverse contexts, incorporating objective metrics, such as global and local drug approval timelines, prescription volumes, and real-world toxicity registries, that can be linked to confidence assessments. Although these data are currently unavailable, collecting them in the future would provide a better understanding of how regulatory environments and clinical exposure shape ADC management expertise.

While our data quantitatively showed specialty differences, we acknowledge that the small sample size of sub-specialties limits the generalizability of our findings. Lastly, although our results suggest a comparable confidence across cancer types, this may not capture differences in drug-specific experience or availability across sub-specialties.

Despite these limitations, our study provides a valuable foundation for understanding global variations in confidence levels for managing ADC toxicities and highlights areas for future surveys and research and targeted interventions in oncology education, training, and practice, eventually improving global competence.

## Supplementary Material

oyaf232_Supplementary_Data

## Data Availability

Data used for this analysis are available upon justified request to the corresponding author.
