# Supplementary material for: Global variations in oncology professionals’ confidence levels for managing antibody–drug conjugate toxicities: a cross-continental survey
Source: Oncologist. 2025 Jul 28;30(9):oyaf232. doi: 10.1093/oncolo/oyaf232 (PMC12445673; doi:10.1093/oncolo/oyaf232)
Supplement: oyaf232_Supplementary_Data [file oyaf232_supplementary_data.docx]

**Supplementary Table 1.** Continent, country income, job role, cancer type and specialization of respondents

| **Continent** | **Countries** |
| --- | --- |
| Africa | Algeria, Angola, Botswana, Cameroon, Egypt, Ethiopia, Gabon, Ghana, Kenya, Libya, Malawi, Mauritius, Morocco, Nigeria, South Africa, Sudan, Tanzania, Tunisia, Zimbabwe |
| Asia | Armenia, Bangladesh, Brunei Darussalam, China, Georgia, Hong Kong, India, Indonesia, Iran, Iraq, Israel, Japan, Jordan, Kazakhstan, Lebanon, Malaysia, Myanmar, Nepal, Oman, Pakistan, Philippines, Saudi Arabia, Singapore, South Korea, Sri Lanka, Thailand, Palestine, State of, Turkey, United Arab Emirates, Vietnam, Yemen, Taiwan |
| Europe | lbania, Austria, Belgium, Bosnia and Herzegovina, Bulgaria, Croatia, Czech Republic, Denmark, Estonia, Finland, France, Germany, Greece, Hungary, Ireland, Italy, Latvia, Lithuania, Luxembourg, Malta, Netherlands, North Macedonia, Norway, Poland, Portugal, Romania, Russian Federation, Serbia, Slovakia, Slovenia, Spain, Sweden, Switzerland, Ukraine, United Kingdom, The Netherlands |
| Oceania | Australia, New Zealand, Papua New Guinea |
| North America | Canada, Costa Rica, Cuba, Dominican Republic, El Salvador, Guatemala, Honduras, Jamaica, Mexico, Panama, Trinidad and Tobago, United States, Belize |
| South America | Argentina, Bolivia, Brazil, Chile, Colombia, Ecuador, Paraguay, Peru, Suriname, Uruguay, Venezuela |
| **Income** | **Countries** |
| High income | Australia, Austria, Belgium, Brunei Darussalam, Canada, Chile, Croatia, Czech Republic, Denmark, Estonia, Finland, France, Germany, Greece, Hong Kong, Hungary, Ireland, Israel, Italy, Japan, Latvia, Lithuania, Luxembourg, Malta, Netherlands, New Zealand, Norway, Portugal, Singapore, Slovakia, Slovenia, South Korea, Spain, Sweden, Switzerland, Taiwan, United Kingdom, United States, Poland, Saudi Arabia, Oman, Trinidad and Tobago |
| Upper-middle income | Albania, Argentina, Armenia, Belize, Bolivia, Bosnia and Herzegovina, Botswana, Brazil, Bulgaria, China, Colombia, Costa Rica, Cuba, Dominican Republic, Ecuador, Georgia, Guyana, Iraq, Kazakhstan, Libya, Malaysia, Mauritius, Mexico, North Macedonia, Paraguay, Peru, Romania, Russian Federation, Serbia, South Africa, Thailand, Turkey, Uruguay, Macedonia, Ukraine |
| Lower-middle income | Algeria, Angola, Bangladesh, Cameroon, Egypt, El Salvador, Gabon, Ghana, Guatemala, Honduras, India, Indonesia, Iran, Jordan, Kenya, Lebanon, Morocco, Myanmar, Nepal, Nigeria, Pakistan, Philippines, Sri Lanka, Tanzania, Tunisia, Vietnam, State of Palestine |
| Low income | Ethiopia, Malawi, Sudan, Zimbabwe |
| Unclassified | Venezuela |
|  |  |
| **Job** | **Roles** |
| Senior physician | Consultant |
| Junior physician | Physician Assistant, Registrar, Resident, Trainee/Fellow |
| Nurse | Nurse |
| Nurse Practitioner | Nurse Practitioner |
| **Cancer type** |  |
| Other | Geriatric, Gynaecologic cancers, Leukemia, Melanoma  Neuro-oncology, Paediatric oncology, Sarcoma |
| **Specialization** |  |
| Haematology | Haematology Nurse, Nurse Practitioner Haematologist-Oncologist, Haematologist Oncologist, Consultant Haematologist, Trainee/Fellow Haematologist, Consultant/Attending Haematologist-Oncologist |
| Medical Oncology | Medical Nurse, Nurse Practitioner Clinical Oncology, Nurse Practitioner Medical Oncology, Consultant Medical Oncologist, Consultant/Attending Medical Oncology, Physician Assistant Medical Oncology, Registrar Medical Oncology, Specialist Registrar Medical Oncology, Trainee/Fellow Medical Oncology |
| Radiation ot Clinical oncology | Radiation Nurse, Clinical Nurse, Radiation Oncologist, Consultant Clinical Oncology, Consultant/Attending Clinical Oncology, Consultant/Attending Radiation Oncology, Registrar Clinical Oncology, Specialist Registrar Clinical Oncology, Trainee/Fellow Clinical Oncology, Trainee/Fellow Radiation Oncology |
| Surgical Oncology | Consultant/Attending Surgical Oncology |

**Supplementary Table 2**. Characteristics of Survey Respondents by specialization

| **Job** | **No.** | **%** |
| --- | --- | --- |
| Nurse | 233 | 15.55 |
| Physician | 1023 | 81.44 |
| **Physician and Nurse speciality** | **No.** | **%** |
| Clinical and Radiation Oncology Nurse | 49 | 3.90 |
| Hematology Nurse | 32 | 2.55 |
| Medical Oncology Nurse | 117 | 9.32 |
| Other Nurse | 35 | 2.79 |
| Clinical and Radiation Oncologist | 332 | 26.43 |
| Hematologist | 48 | 3.82 |
| Medical Oncologist | 580 | 46.18 |
| Surgeon | 38 | 3.03 |
| Other | 25 | 1.99 |
| **Physician role** | **No.** | **%** |
| Senior physician | 548 | 53.57 |
| Junior physicians | 475 | 46.63 |
| **Nurse role** | **No.** | **%** |
| Nurse | 163 | 69.95 |
| Nurse Practitioner | 70 | 30.04 |

**Supplementary Table 3.** Characteristics of Survey Respondents by Profession and Cancer Type treated.

|  | **Physician (1023)** | | **Nurse (233)** | | **Total (1254)** | |
| --- | --- | --- | --- | --- | --- | --- |
| **Cancer type** | **No.** | **%** | **No.** | **%** | **No.** | **%** |
| Breast cancer | 310 | 30.30 | 75 | 32.19 | 385 | 30.65 |
| Gastrointestinal cancers | 388 | 37.93 | 75 | 32.19 | 463 | 36.86 |
| Genitourinary cancers | 33 | 3.23 | 7 | 3.00 | 40 | 3.18 |
| Geriatric | 8 | 0.78 | 4 | 1.72 | 12 | 0.96 |
| Gynaecologic cancers | 23 | 2.25 | 9 | 3.86 | 32 | 2.55 |
| Head and Neck cancer | 62 | 6.06 | 6 | 2.58 | 68 | 5.41 |
| Leukemia | 21 | 2.05 | 7 | 3.00 | 28 | 2.23 |
| Lung cancer | 115 | 11.24 | 18 | 7.73 | 133 | 10.59 |
| Lymphoma | 24 | 2.35 | 13 | 5.58 | 37 | 2.95 |
| Melanoma | 13 | 1.27 | 8 | 3.43 | 21 | 1.67 |
| Neuro-oncology | 11 | 1.08 | 5 | 2.15 | 16 | 1.27 |
| Paediatric oncology | 2 | 0.20 | 2 | 0.86 | 2 | 0.32 |
| Sarcoma | 12 | 1.17 | 2 | 0.86 | 14 | 1.11 |
| NA | 1 | 0.10 | 2 | 0.86 | 3 | 0.40 |

**Supplementary Table 4**. Characteristics of Survey Respondents by Country Income Level.

|  | **Physician** | | **Nurse** | | **Total** | |
| --- | --- | --- | --- | --- | --- | --- |
| **Income Level** | **No.** | **%** | **No.** | **%** | **No.** | **%** |
| Low Income | 7 | 0.68 | 2 | 0.86 | 9 | 0.72 |
| Lower-middle income | 291 | 28.45 | 11 | 4.72 | 302 | 24.04 |
| Upper-middle-income | 335 | 32.75 | 20 | 8.58 | 355 | 28.26 |
| High income | 390 | 38.12 | 200 | 85.84 | 590 | 46.97 |

**Supplementary Table 5.** Characteristics of Survey Respondents by Country

|  | **Physician** | | **Nurse** | | **Total** | |
| --- | --- | --- | --- | --- | --- | --- |
| **Continent** | **No.** | **%** | **No.** | **%** | **No.** | **%** |
| Africa | 79 | 7.72 | 5 | 2.15 | 84 | 6.69 |
| Asia | 326 | 31.87 | 15 | 6.44 | 341 | 27.15 |
| Europe | 349 | 34.12 | 115 | 49.36 | 464 | 36.94 |
| North America | 94 | 9.19 | 70 | 30.04 | 164 | 13.06 |
| South America | 156 | 15.25 | 19 | 8.15 | 175 | 13.93 |
| Oceania | 19 | 1.86 | 9 | 3.86 | 28 | 2.23 |

**Supplementary Table 6.** Confidence in Management Toxicities Among Nurses by Continent.

|  | **Continent** | | | | | | | | | | |
| --- | --- | --- | --- | --- | --- | --- | --- | --- | --- | --- | --- |
| **Confidence** | **Africa (5)** | **%** | **Asia**  **(15)** | **%** | **Europe (115)** | **%** | **America (89)** | **%** | **Oceania**  **(9)** | **%** | **p-value** |
| 1 | 0 | 0.00 | 3 | 20.00 | 13 | 11.30 | 4 | 4.49 | 3 | 33.33 |  |
| 2 | 0 | 0.00 | 0 | 0.00 | 13 | 11.30 | 4 | 4.49 | 1 | 11.11 | *p=***0.02** |
| 3 | 1 | 20.00 | 3 | 20.00 | 24 | 20.87 | 29 | 32.58 | 0 | 0.00 |  |
| 4 | 2 | 40.00 | 1 | 6.67 | 36 | 31.30 | 19 | 21.34 | 2 | 22.22 |  |
| 5 | 2 | 40.00 | 8 | 53.33 | 29 | 25.22 | 33 | 37.07 | 3 | 33.33 |  |

**Supplementary Table 7.** Confidence in Management Toxicities Among Nurses by Country Income Level.

|  | **Income level** | | | | | | | | | | |
| --- | --- | --- | --- | --- | --- | --- | --- | --- | --- | --- | --- |
| **Confidence** | **Low**  **(2)** | **%** | **Lower-middle**  **(11)** | **%** | **Upper-middle**  **(20)** | **%** | **High**  **(200)** | **%** |  |  |  |
| 1 | 0 | 0.00 | 3 | 27.27 | 0 | 0.00 | 20 | 10.00 |  |  |  |
| 2 | 0 | 0.00 | 0 | 0.00 | 0 | 0.00 | 18 | 9.00 |  |  | *p<***0.001** |
| 3 | 0 | 0.00 | 1 | 9.09 | 4 | 20.00 | 52 | 26.00 |  |  |  |
| 4 | 1 | 50.00 | 0 | 0.00 | 2 | 10.00 | 57 | 28.50 |  |  |  |
| 5 | 1 | 50.00 | 7 | 63.64 | 14 | 70.00 | 53 | 26.50 |  |  |  |

**Supplementary Table 8.** Confidence in Management Toxicities Among Nurses.

| **Confidence** | **Nurse** | **%** | **Nurse Practitioner** | **%** | **p-value** |
| --- | --- | --- | --- | --- | --- |
| 1 | 13 | 7.97 | 10 | 14.28 |  |
| 2 | 15 | 9.2 | 3 | 4.28 |  |
| 3 | 36 | 22.08 | 21 | 30 | *p=*0.11 |
| 4 | 41 | 25.15 | 19 | 27.14 |  |
| 5 | 58 | 35.58 | 17 | 24.29 |  |

**Supplementary Table 9.** Confidence in Management Toxicities Among Survey Respondents by job.

| **Confidence** | **Physician** | **%** | **Nurse** | **%** | **p-value** |
| --- | --- | --- | --- | --- | --- |
| 1 | 85 | 8.31 | 23 | 9.87 |  |
| 2 | 49 | 4.79 | 18 | 7.73 |  |
| 3 | 253 | 24.73 | 57 | 24.46 | *p=*0.77 |
| 4 | 264 | 25.81 | 60 | 25.75 |  |
| 5 | 372 | 36.36 | 75 | 32.19 |  |

**Supplementary Table 10.** Confidence in Management Toxicities Among Survey Respondents by Nurse Specialization.

| **Nurse speciality** | | | | | | | | | |  |  |
| --- | --- | --- | --- | --- | --- | --- | --- | --- | --- | --- | --- |
| **Confidence** | **Clinical and Radiation** | **%** | **Haematology** | **%** | **Medical** | **%** | **Other** | **%** | **p-value** |  |  |
| 1 | 5 | 10.20 | 4 | 12.5 | 8 | 6.84 | 6 | 17.14 |  |  |  |
| 2 | 2 | 4.08 | 3 | 9.38 | 11 | 9.40 | 2 | 5.71 |  |  |  |
| 3 | 14 | 28.57 | 7 | 21.87 | 28 | 23.93 | 8 | 22.86 | *p=*0.79 |  |  |
| 4 | 17 | 34.69 | 8 | 25 | 29 | 24.79 | 6 | 17.14 |  |  |  |
| 5 | 11 | 22.45 | 10 | 31.25 | 41 | 35.04 | 13 | 37.14 |  |  |  |

**Supplementary Table 11.** Confidence in Management Toxicities by cancer type. “Other”: aggregated tumor types with less than 30 observations.

|  | **Cancer type** | | | | | | | | | | | | | | | |  | |  | |
| --- | --- | --- | --- | --- | --- | --- | --- | --- | --- | --- | --- | --- | --- | --- | --- | --- | --- | --- | --- | --- |
|  | **Breast** | **%** | **Gastrointestinal** | **%** | **Genitourinary** | **%** | **Gynaecologic** | **%** | **Head&Neck** | **%** | **Lung** | **%** | **Lymphoma** | **%** | **Other** | **%** | | **p-value** | |  |
| **Confidence** |  |  |  |  |  |  |  |  |  |  |  |  |  |  |  |  | |  | |  |
| **1** | 33 | 8.57 | 34 | 7.34 | 3 | 7.50 | 3 | 9.38 | 11 | 16.18 | 11 | 8.27 | 3 | 8.11 | 10 | 10.31 | |  | |  |
| **2** | 16 | 4.16 | 26 | 5.62 | 4 | 10.00 | 2 | 6.25 | 3 | 4.41 | 7 | 5.26 | 4 | 10.81 | 5 | 5.15 | | *p=*0.44 | |  |
| **3** | 103 | 26.75 | 107 | 23.11 | 7 | 17.50 | 5 | 15.63 | 21 | 30.88 | 28 | 21.05 | 12 | 32.43 | 27 | 27.84 | |  | |  |
| **4** | 98 | 25.45 | 121 | 26.13 | 13 | 32.50 | 11 | 34.38 | 16 | 23.53 | 40 | 30.08 | 7 | 18.92 | 18 | 18.56 | |  | |  |
| **5** | 135 | 35.06 | 175 | 37.80 | 13 | 32.50 | 11 | 34.38 | 17 | 25.00 | 47 | 35.34 | 11 | 29.73 | 37 | 38.14 | |  | |  |

**Supplementary Figure 1**. ONCOassist characteristics.


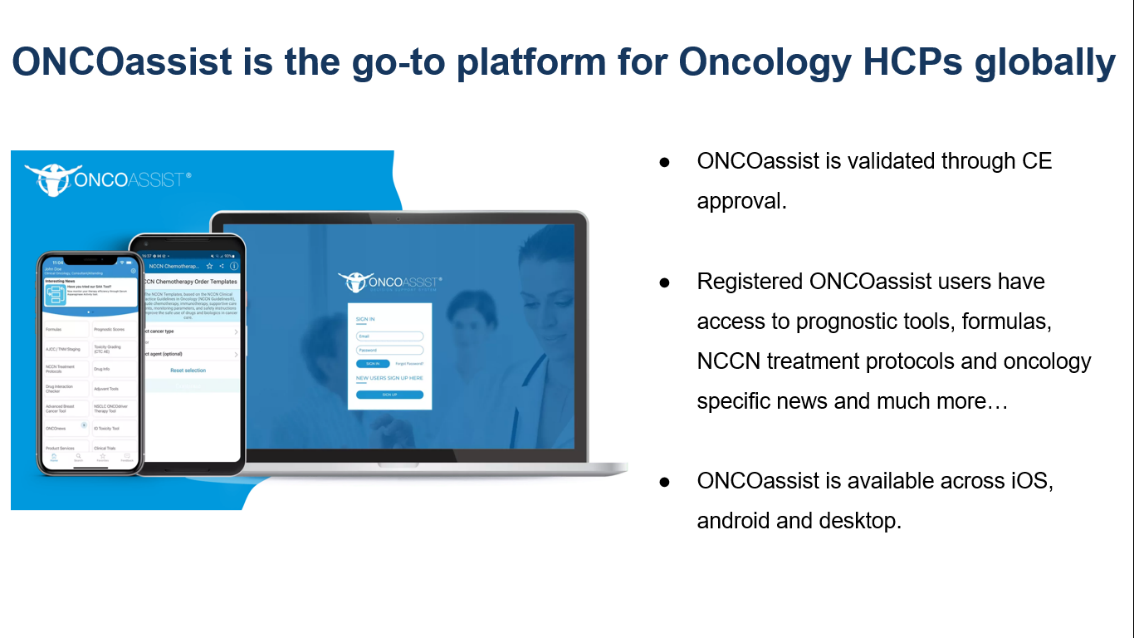


**Supplementary Figure 2.** The question.


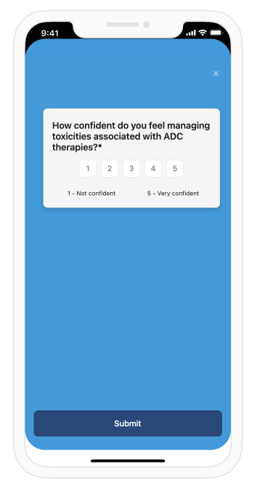


**Supplementary Figure 3**. ONCOassist Registration process.


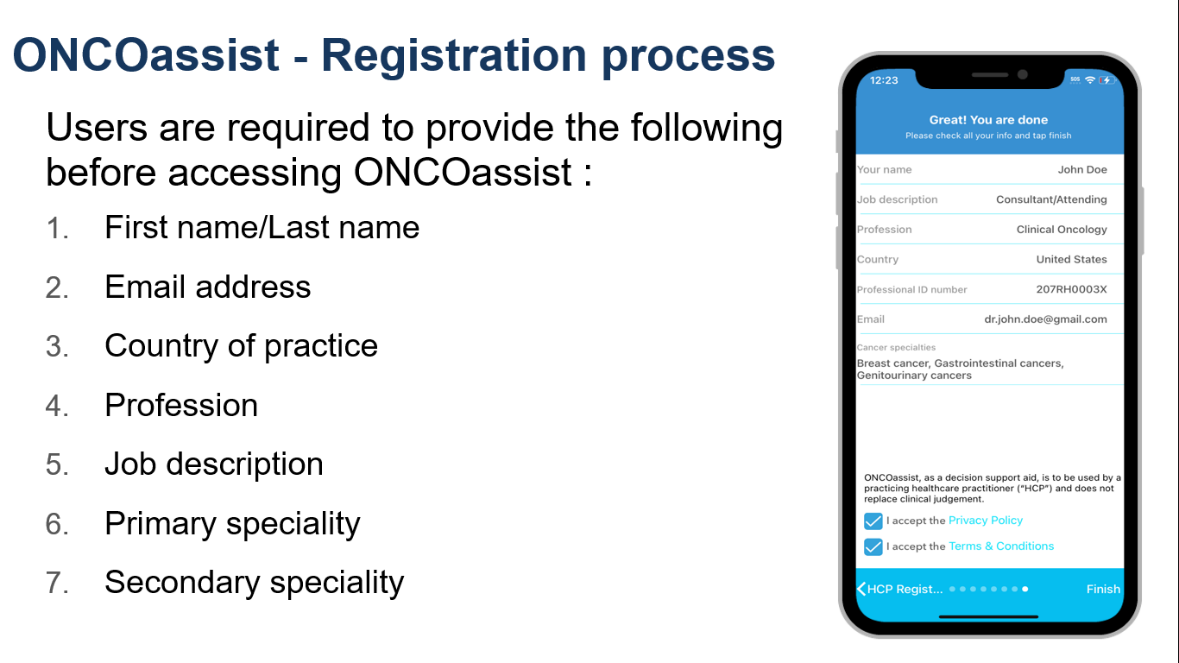


**Supplementary Figure 4.** Confidence in Management Toxicities Among Survey Respondents by Country.


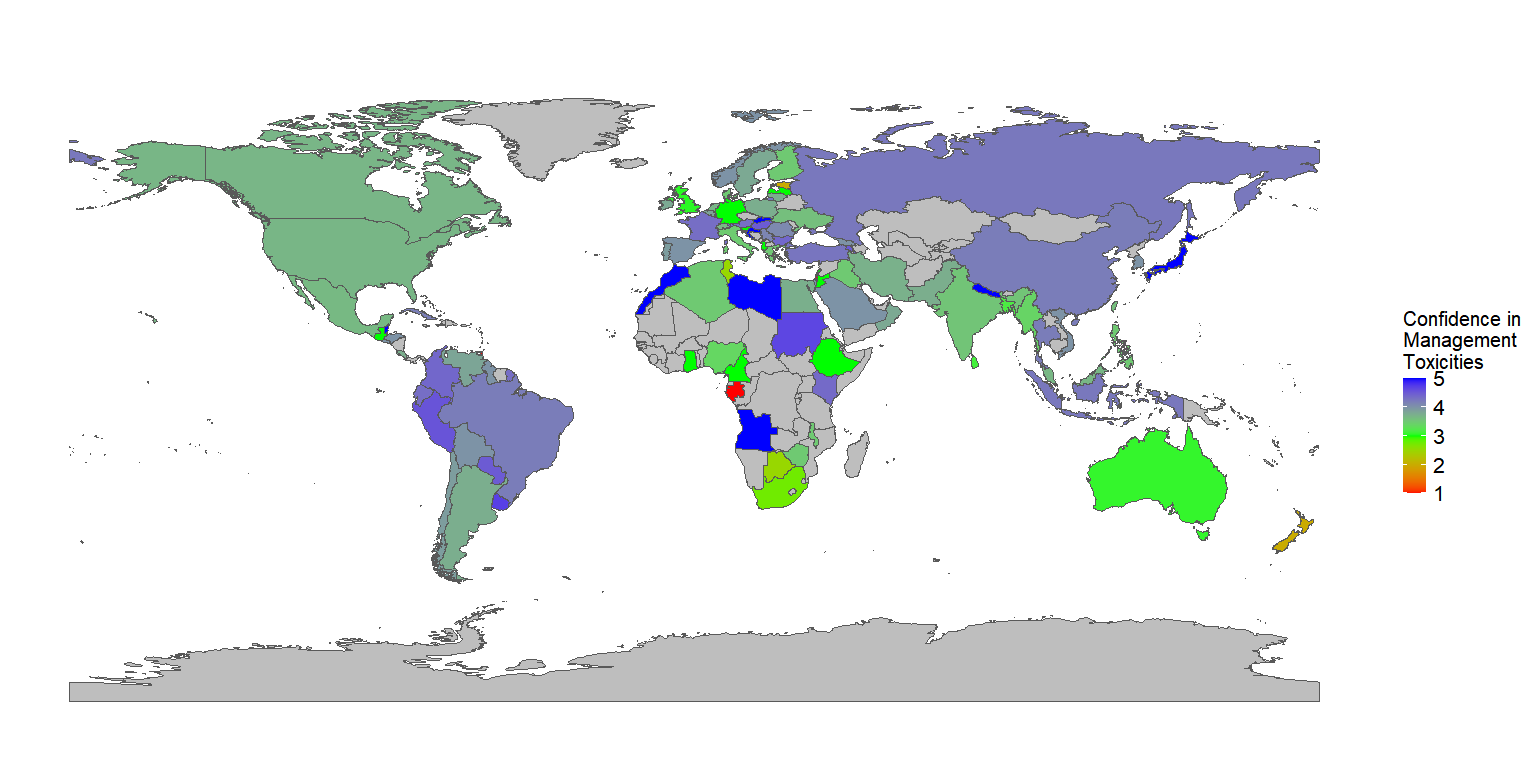


**Legend**: World map illustrating the levels of confidence in managing ADC toxicities across various countries. Countries are color-coded based on confidence levels, with a gradient from red (indicating the lowest confidence, level 1) to dark blue (indicating the highest confidence, level 5). Countries shaded in grey represent regions with no available data.
